# Application of Interprofessional Education Model to University Pre-Licensure Health Students in the Management of Chronic Care Conditions in Zambia

**DOI:** 10.55320/mjz.49.2.1118

**Published:** 2022

**Authors:** Emmanuel M. Musenge, Selestine H. Nzala, Marjorie Kabinga-Makukula, Ruth Wahila, Penelope Machona, Aubrey C. Kalungia, Harrison Daka, Micah Simpamba, Mercy M. Imakando, Violet Kayamba, Victoria Mwiinga-Kalusopa, George Soko, Moses C. Simuyemba, Linda Kampata-Olowski, Cosmas Zyambo, Patricia Katowa-Mukwato, Micheal Chigunta, Masauso M. Phiri, Christabell Mwiinga, Concepta Kwaleyela, Trevor Kaile, Bellington Vwalika, Elliot Kafumukache, Margaret C. Maimbolwa, Fastone M. Goma

**Affiliations:** 1 School of Nursing Sciences, University of Zambia, Lusaka, Zambia,; 2 School of Medicine, University of Zambia, Lusaka, Zambia,; 3 School of Health Sciences, University of Zambia, Lusaka, Zambia,; 4 Women and Newborn Hospital, University Teaching Hospitals, Lusaka, Zambia,; 5 Adult Hospital, University Teaching Hospitals, Lusaka, Zambia,; 6 School of Public Health, University of Zambia, Lusaka, Zambia,; 7 School of Medicine and Health Sciences, Mulungushi University, Zambia

**Keywords:** Interprofessional Education, Interprofessional Collaboration, Multidisciplinary Health Care Teams, Chronic Care Conditions, Training Modules

## Abstract

**Background:**

There is evidence that multidisciplinary healthcare teams can provide better quality of care and treatment outcomes compared to that delivered by individuals from a single health discipline. The project on which this article is based applied the interprofessional education model to university pre-licensure health students in the management of chronic care conditions in Zambia.

**Methods:**

Four distinct but interrelated approaches, namely desk review; module development workshops; review and validation of modules by experts; piloting and review of the training modules were employed.

**Results:**

Several models of interprofessional education currently in existence and used successfully by higher education institutions in other settings were identified. While several models of Interprofessional Education were identified, our project adapted the “didactic program, community-based experience, and interprofessional-simulation experience” models. To apply the models, modules of seven chronic care conditions were developed and piloted. The extent to which the module activities promoted interprofessional education were rated between 74 – 87% (agree or strongly agree) by the students.

**Conclusion:**

Three models of Interprofessional Education were identified and adapted in the project, and seven modules were developed and administered to the students. The process was effective for putting forth an interprofessional training program at the undergraduate level, with the potential to improve quality of care for patients.

## INTRODUCTION

Globally, non-communicable and communicable chronic diseases are leading causes of morbidity and mortality^1^, especially in low- and middle-income country settings of sub-Saharan Africa.^2^ A growing body of evidence shows that educational interventions can improve the health professionals’ capacity of detecting and managing chronic diseases.^3,4^ Multidisciplinary healthcare teams can provide better treatment outcomes and quality of care compared to that delivered by individual professionals from a single health discipline.^5,6^ This places great importance on interprofessional education (IPE) approaches to chronic conditions management and patient care.

Interprofessional education is an experience that occurs when students from two or more professions learn about, from, and with each other.^7^ In health professions training programmes offered across countries, the ultimate goal of IPE is to catalyze transformation in healthcare training and foster continued collaboration among healthcare professionals.^8^ IPE that encompasses actual and/or simulated clinical practice allows for higher levels of learning, such as changes in behavior and improved performance at both the level of the individual health professional and the healthcare team.^6^ Embedding Interprofessional Learning (IPL) within core curricula remains a significant challenge in many settings.^9,10,11^ Arguably, this is particularly challenging for health training programmes whose curricula, disciplines, educational philosophies and strategies may vary significantly.

Whereas the need and importance of IPE have been widely described elsewhere^12,7^, the process of curriculum development for IPE courses or modules has not been adequately documented globally in the literature for use by educators planning such educational interventions.^13^ In 2019, the Strengthening Health Professional Workforce Education Programmes for Improved Quality Health Outcomes in Zambia (SHEPIZ) project embarked on, among other aims, developing IPE in chronic conditions management. The intended outcomes, as guided by the best practice model^14^, were premised on enhancing collaborative practice which could lead to strengthened health systems and optimal health service delivery in Zambia. In this paper, we present the process of developing and piloting IPE modules on chronic conditions management for the undergraduate health professions training curriculum in Zambia.

## METHODS

The methodology adopted in the project can be structured into four distinct but interrelated approaches: (1) Desk review, (2) Module development workshops (3) Expert review of IPE modules on chronic conditions and; (4) Piloting and review of the IPE training modules. The project obtained ethical approval from the University of Zambia Biomedical Research Ethics Committee on reference number 920–2020.

### Desk Review

**Figure 1:**
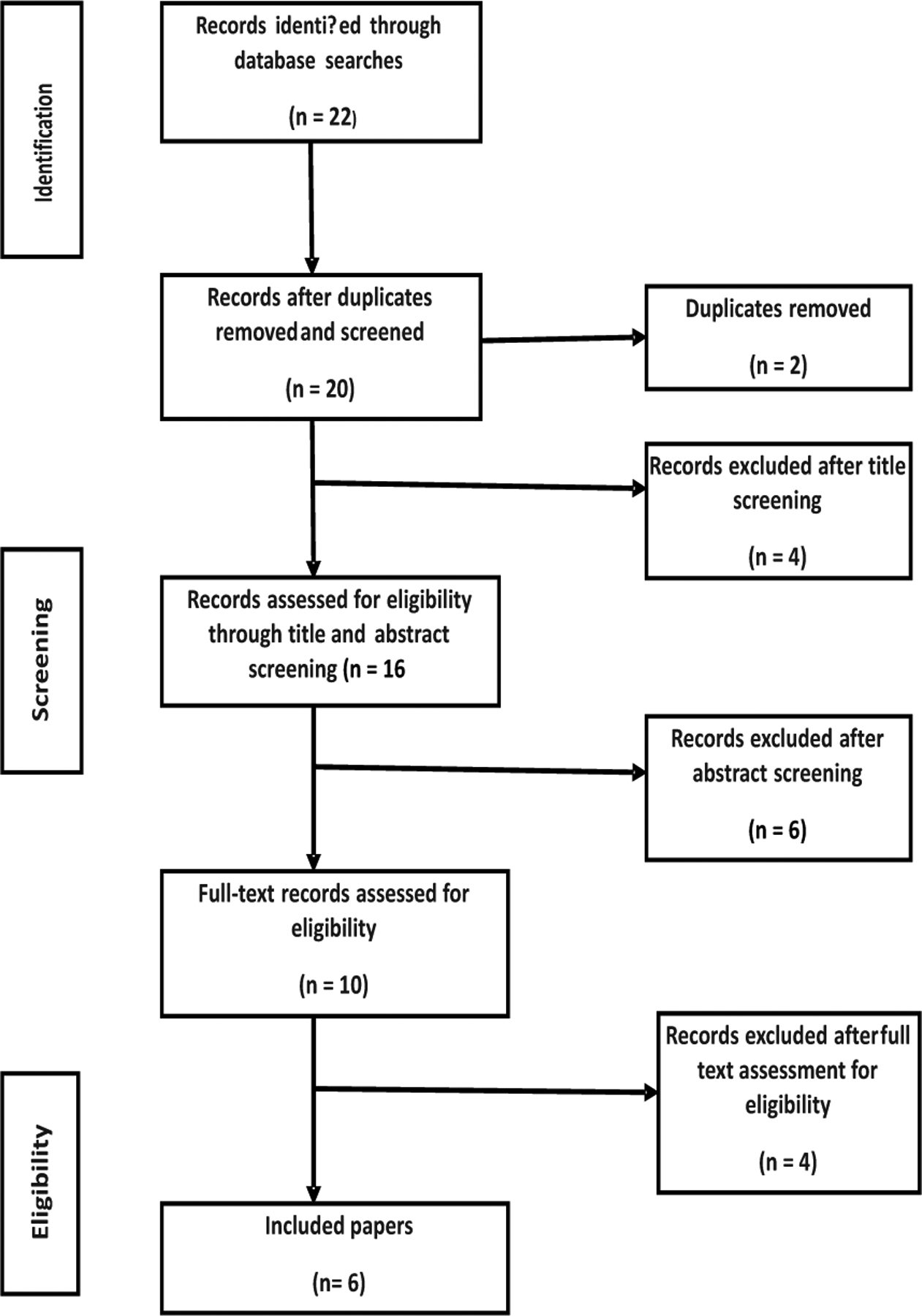
Flowchart for the Desk Review

From September to December, 2019 a desk review was conducted to identify best practices in IPE focusing on models of IPE, competences, content, training materials, delivery methods, and assessment methods. Literature search was conducted by three research experts; one from the University of Alabama and two from the University of Zambia (UNZA). Google Scholar and PubMed electronic databases were searched for articles written in English and published from 1^st^ January, 2009 to 31^st^ October, 2019, using the terms, “interprofessional education, interprofessional learning, interprofessional collaboration (IPC), interprofessional practice,” combined with “chronic diseases and undergraduate health professional students.” These publication years were chosen because we intended to identify the latest approaches to IPE. The inclusion criteria for this review were data-based, health profession-related, research articles with terms such as interprofessional education, interprofessional learning, IPC, interprofessional practice in their titles or abstracts as well as in the main texts of the publications. Titles and abstracts were then reviewed by 12 participants to determine if the publication met the inclusion criteria. All articles meeting the inclusion criteria were then read independently in full by the same 12 participants who reviewed the titles and abstracts to determine if the terms “interprofessional education, interprofessional learning, IPC, interprofessional practice” were clearly stated in the main texts. Any articles in which authors did not precisely state these key terms in the main text were excluded. A total of 22 articles met the inclusion criteria (Figure 1). The independent review process took within 14 days to complete.

A checklist was developed through consensus building among participants to help extract information from the documents. The process of checklist development was adapted from^15^ guidelines for developing evaluation checklists. Participants were engaged through a one-day meeting to clarify and justify the criteria to be met by the checklist. The documents were deemed to be relevant based on their focus on the project which is IPE for chronic conditions and their applicability to the Zambian situation. Further, the journal titles in which the documents were published were searched in **Ulrichs International Periodicals Directory** and only articles from scholarly journals were considered to be relevant. The checklist was then applied for its intended purpose of extracting relevant information on models that are relevant to the Zambian situation in implementing IPE in teaching chronic conditions. Reading through the documents identified for review, six were identified to be relevant to the Project.^14,16,17,18,19,20^

In addition, recognized reference documents for guidelines on treatment and management of chronic conditions were identified through **author name, date of publication, title of the work, and publication data**. Both local international and local guidelines were considered. Some of the documents that were identified were treatment and management guidelines from the Zambian Ministry of Health, professional associations such as American Diabetic Association, United Nations such as **Joint United Nations Programme on HIV/AIDS** (UNAIDS) and World Health Organisation.

### Workshops to develop training modules for chronic conditions

A technical working group comprising 10 members was formed and composed of diverse experts with experiences on IPE, medical education, general education, and management of chronic diseases. Three workshops were held with members of the technical working group as part of the process of developing modules for chronic conditions. The workshops were held between July 2020 and March 2021. During the workshops, one participant was selected to be a moderator responsible for particular parts of the workshop on different days. A collaborative approach was used where the lead and co-lead persons and participants worked together through the content of the modules. In all workshops, an in-service training format was adopted, using guided discussions, presentations, peer review and plenary to refine the module content.

The first workshop started the module development process and facilitated discussions on identification and building consensus on the common chronic conditions currently prevailing in Zambia which was guided by the priority health problems listed by the Ministry of Health. Seven chronic care conditions were identified and selected including hypertension, diabetes mellitus, stroke, human immunodeficiency virus (HIV), pulmonary tuberculosis (PTB), cervical and prostate cancers.

The module writing workshop followed the five steps of writing although the process was not linear. The first step involved brainstorming, focusing and developing a frame for module writing. Workshop participants shared their experiences on module writing before agreeing to the standard format. In addition, workshop participants reviewed the critical sections of the modules such as phrasing of objectives and developing case scenarios. Facilitation of these critical sections was conducted by two participants based on their expertise in education for health professionals. In the second step, a search for sources of information for each chronic condition was conducted and these sources were listed. In the third step, development of the draft module was completed. This was followed by the fourth step whose main focus was revision of the draft modules. The revision was facilitated by periodic evaluation of the content in terms of correct information, clarity and flow of information. The evaluation process was conducted through presentations, guided discussions, plenary sessions and individual consultations with other workshop participants and subject experts from the University Teaching Hospitals (UTH). The number of subject experts from UTH were seven; corresponding to the total number of modules. All the subject experts were senior medical practitioners specialized in managing the chronic conditions assigned to and were actively practicing. The last step involved proofreading and editing.

The other two workshops were follow-up meetings held to review the technical aspects of developing and further standardising the design of the modules. All the 10 participants from the technical working group participated in the two follow-up workshops. During one of the workshops, the module development team were trained on curriculum and case relations development by an expert from Vanderbilt University, United States; the information which further guided the development of case simulations and standardized patient approaches for chronic conditions. Further, the developers of the modules reviewed the comments from the subject experts on each module to improve the content of the modules and their delivery modes.

The modules were developed in two parts, initially, the facilitator’s module on each chronic condition was developed after which the participant’s module was then mirrored to include activities for the participants. The participant modules were designed to be interactive with full participation during the delivery of each module using various types of activities that were case-based. Case simulations were utilised to ensure the participation of the learners during the training. After module writing was completed, all the seven modules were printed in readiness for the pilot training.

### Expert review of IPE modules on chronic care conditions

The team comprised professionals from the fields of medicine, nursing, pharmacy, physiotherapy and education to allow for multidisciplinary health care teaching and learning. The modules were reviewed by carefully selected experts in the relevant fields with vast experience in managing the specific chronic conditions. The experts were selected from Zambia’s highest tertiary institution, and local research and training institutions. The experts’ comments were sent to the technical working group who incorporated the comments taking into consideration the level of depth and relevance to the target group of learners. The expert review process was undertaken concurrently with the workshops to develop the IPE modules on chronic conditions.

### Piloting and review of the IPE modules on chronic care conditions

To finalise the development of the modules, a 3-day interprofessional pilot training workshop involving Medical, Nursing and Physiotherapy undergraduate students was conducted in November 2020. The process of conducting the pilot served as a mutual interprofessional learning platform that will guide future training. At the same time, it gave the project team further insights on the module content and process of delivery through the student evaluation report. Likert scales were used to assess the views and attitudes of the students towards the modules and the pilot implementation. This was followed by a thematic analysis of responses. Pilot facilitators, who were independent people that were not part of the development of the modules gave their own observations and feedback. The pilot training was followed by another workshop in March 2021 that incorporated recommendations from students who participated in the pilot training.

## FINDINGS AND DISCUSSION

To the best of our knowledge, the development of University-based IPE modules on care of chronic conditions specifically tailored to the context of Zambia is novel. Students trained using an IPE approach are more likely to become collaborative interprofessional team members who show respect and positive attitudes towards each other and work towards improving patient outcomes.^21^

### Desk review

#### Relevant models that were adapted

The desk review, showed that there were best practices that could be relevant to IPE at UNZA in relation to the use of IPE for undergraduate programmes. Some models focus on the integration of patient involvement as part of team practice while others focus on decision making cooperation between professionals and coordination of members of the team during tasks.^14,16,17,18,19,20^

While several models of IPE exist, our project adapted the “didactic program, community-based experience, and interprofessional-simulation experience” models.^14^ The didactic program emphasizes interprofessional team building skills, knowledge of professions, patient centered care, service learning, the impact of culture on healthcare delivery and an interprofessional clinical component. The community-based experience demonstrates how IPCs provide service to patients and how the environment and availability of resources impact one’s health status. The interprofessional-simulation experience describes clinical team skills training in both formative and summative simulations used to develop skills in communication and leadership.^14^ This assisted students in understanding their own professional identity while gaining an understanding of other professional’s roles on the health care team. During IPE, students focus on a collaborative approach to patient-centered care, with emphasis on team interaction, communication, service learning, evidence-based practice, and quality improvement.^14^

#### Overarching competencies identified

This project adapted six competence domains for interprofessional collaborative practice.^16^ The six competence domains include interprofessional communication, client-centered care, role clarification, team functioning, collaborative leadership, and interprofessional conflict resolution. Other institutions have identified similar competencies for IPE. The Interprofessional Education Collaborative [IPEC]^18^ identified four competency domains, namely values for interprofessional practice, roles and responsibilities for collaborative practice, interprofessional communication practices, and interprofessional teamwork and team-based practice. The Canadian National Interprofessional Competency Framework provides an integrative approach to describing the competencies required for effective IPC.^16^ The competency domains highlight the knowledge, skills, attitudes and values that shape the judgments essential for interprofessional collaborative practice.

#### Content

Professional commonalities should be considered in the formulation of the content. Professors of the professions involved in the program should participate in content development. It should be learner-based, profession-based, and patient-based care and cover all educational goals. Adult education should be problem-centered and function oriented, rather than content-centered. Using adult learning principles and best practices open the door for student creativity, hands on learning and success of the teaching and learning process.^22^

#### Delivery methods

The common delivery methods from literature include service learning projects, mentorship, didactic presentations which can be face-to-face in workshops or online using available learning management systems such as Moodle, role model demonstrations of clinical supervisors and simulations.^14^ Students who attended an active learning activity show increased interprofessional teamwork and team-based practice, roles/ responsibilities for collaborative practice, and better patient outcomes from collaborative practice. Our project demonstrates the utility of an IPE on student and highlights the potential importance of active interprofessional learning offerings.^23^

#### Learning strategies

The learning strategies should be interactive, learner-based, and patient centered, using problem-based learning methods. The methods should create learning synergy, share experiences, and be implemented in formative-summative method. Some teaching and learning strategies used in other settings include seminars, workshops, small group discussions, role-playing exercises, clinical round discussions, journal clubs, simulations, team case conferences, clinical placement with clients. These strategies provide an opportunity to train healthcare students in a safe environment through observation, hands-on training, team interaction and critical feedback.^24^ It is clear that these strategies of IPE modify the attitudes of prospective healthcare professionals by exposing them to interactive communication, mutual respect and teamwork, thus facilitating the adoption of IPC in healthcare settings.^25,26^

#### Assessment methods

The evaluation of the students should be based on the goals and competencies. From the Ontario post-registration IPE model, assessment is based on George Miller’sclinical education framework to show the movement of a student from the entrance to their pre-registration program through to their completion.^27^ Common assessment methods include knowledge, attitude, and practice questionnaires; clinical context tests such as exams, quizzes, essays, oral; self-reflection, reflective journaling, professional portfolios; clinical placement evaluation; feedback from clients; Objective Structured Clinical Examination (OSCE); behaviour rating scales; video audits; peer feedback; online discussion rating and group presentations. Despite progress made in IPE, there remains substantial difficulty in considerable variability in assessment of learners’ interprofessional collaborative knowledge and skills. There is need to use these assessment tools with an explicit program-evaluation frame-work. For IPE to advance and to align with the demands of changing clinical care systems, robust assessment and evaluation methods, standardized use of common tools, and longitudinal assessment from diverse data streams are needed.^28^

#### Resources critical to achieving Interprofessional Education

Several resources are required for the successful implementation of IPE. Critical resources include Interprofessional faculty members who help in facilitating the implementation of the programme and mentoring the students. When planning for IPE, there is need to educate interdisciplinary faculty members about the need for IPE.^29^ Evidence has stressed the importance of preparing faculty members for IPE including the use of formal course work.^30^ This encourages dialogue for IPCs among health professions programs and assist in identifying opportunities for IPE.^31^

#### Challenges that need mitigation

- There are resistances to implementing interprofessional training programs among different professionals.
- Planning and coordinating the implementation of these programs may be challenging because of the differences in the culture of individuals and conflicts of interest.
- The lack of formal and academic experience in IPE and not being familiar with interprofessional training among the faculty.

### Development, Expert review and validation, and Piloting and review of Modules

The seven modules were selected due to the double burden of communicable and non-communicable diseases plaguing the Zambian health care system. Our country is affected by delayed epidemiologic transition, characteristic of most developing countries.^32^ Cervical cancer is the most prevalent cancer among women and the highest cause of cancer-related mortality in females. Prostate cancer is the commonest cancer among males and overall third commonest cause of cancer-related morbidity.^33^ HIV-related conditions, PTB, Diabetes Mellitus, Stroke and Hypertension are among the leading causes of mortality and morbidity in Zambia.^34,35^

The process of developing the modules for IPE focused mainly on curricula mechanisms such as program content, shared objectives, adult learning principles, learning methods, contextual learning, logistics and scheduling. To a lesser extent, it also focused on educator mechanism, namely staff training, champions, institutional support, managerial commitment and learning outcomes.^7^ Evidence-based practice IPE modules have been proved to improve allied health students’ confidence and knowledge.^36^ IPE in healthcare is being considered as a key factor in providing patient-centred, responsive and high-quality care.^37^

Once the IPE modules were developed, they underwent thorough scrutiny by experts in the relevant fields. Corrections and adjustments were effected accordingly to improve the modules and ultimately improve the teaching and learning experience of the students. Clinical leadership can play pivotal role in connecting the IPE to interprofessional practice. Scientifically crafted clinical faculty development program and overcoming the resistance across incurred by departmental barriers can prepare the practicing practitioners for effective delivery of IPE in improving quality of patient care.^38^ The successful implementation of IPE module supported by expert faculty and sufficient resources carries a strong promise of enhancing its effectiveness with consequent improved patient care and safety.^36^

Finally, the modules were piloted among medical, Nursing and Physiotherapy students. Overall, piloting the modules achieved key objectives. The pilot revealed that students were able to identify the need to work together with other health professionals and they also acknowledged that this was key in provision of care to patients with chronic diseases. Most students (90%) felt that the modules were well organized, relevant and applicable in undergraduate training. The extent to which the activities promoted IPE were rated between 64% and 87% although a few students felt that the modules were somewhat biased towards medical aspects and to a less extent nursing and physiotherapy. These findings reflect the need for continued medical education in IPE both to pre-licensure and licensed health Professionals to continually break down stereotypes preventing teamwork and promote interprofessional respect.^39,40,36^

#### Successes and Challenges during module development

##### Successes

The process built capacity for Interprofessional education and module development, accorded a hands-on experience of the feasibility and benefits of IPC as a better and more holistic approach to patient care. Consequently, an atmosphere of mutual respect and value for other professions was built, in essence igniting a shift in paradigm from profession centrism to a culture of inter-professionalism.^41^ Multidisciplinary input at all levels of module creation culminated in content suitable for students from all the disciplines, while inherently warranting participation from the different professions for successful completion. The module developers themselves had to overcome several barriers to IPE to create modules that effectively promote IPC in patient care settings.

##### Challenges

Several challenges were noted throughout the pilot. Most importantly, the time allocated was grossly inadequate. The five-day program was compressed into only three days. This affected the roll-out of some of the modules and the majority of activities could not be carried out. Financial constraints contributed to a reduction on the days for the pilot. Financial challenges are recognized barriers to the effective implementation of IPE programs.^42^ Innovative ways of overcoming this bottleneck will be key in rolling out the program in full swing in the various schools. Coupled with this, many aired out the need for pre-reading of the modules which should be availed to all the students beforehand to enable more meaningful participation. Additionally, the timing of the pilot was towards exam time for some of the students. This meant divided attention. Synchronizing calendars for the different disciplines of students is a recognized challenge to IPE.^43^ The beginning of the academic year or the period immediately after exams were proposed as a more suitable time for effecting the modular activities. Besides, students felt that there should be a form of reward system, most importantly certificates of attendance and to a lesser extent, an allowance of appreciation for contributing to the modules.

Other logistical challenges included, poor internet connectivity at the venue and lack of electronic visual aids. There were also few facilitators for the program. Since there was no training of facilitators as earlier planned, the developers had to take up the role. By implication, additional views on the content and user-friendliness of the modules from the facilitators other than developers were lacking and a possible source of bias.

##### Recommendations

Learning from the present experience, the following are recommendations for enhancing the success of the process:

- Prioritise what is to be taught through IPE approaches in resource limited settings based on disease burden and need
- Involve students from different professions in the development process for IPE learning materials
- Train facilitators for the module implementation
- Time to whatever extent possible, for the program to be rolled out as far from exam times as possible.
- Innovative interventions for sustainable mobilization of resources to have the training in the community setting, for the correct duration, adequate human resource and medical equipment to facilitate simulations
- There is need to include a clinical component to complete the circuit of stakeholders involved in the IPE for our students

## LIMITATIONS

The project was unable to assess the impact of IPE on learners using higher-level educational outcomes, especially changes in observable behavior due to the short period of application of the models. Considering the fact that there is great need for evidence in this area, the project intends to address this need in future.

## CONCLUSION

The process of developing chronic conditions modules involved four distinct but interrelated approaches. Modules for Hypertension, Diabetes Mellitus, Stroke, HIV, PTB, Cervical Cancer and Prostate Cancer were successfully developed and piloted among Medical, Nursing and Physiotherapy students. Considering all the findings, the best fit model for implementing IPE for chronic care conditions at UNZA undergraduate program level appears to be the “didactic program, community-based experience, and interprofessional-simulation experience” models. The process puts forth an Interprofessional training program at the undergraduate level, with the potential to improve the quality of care and safety for patients with chronic conditions. Despite being fraught with many challenges, the process ultimately was a huge leap in the right direction and turning point for medical education in Zambia.

## What is already known on this topic

- Educational interventions can improve the health professionals’ capacity of detecting and managing chronic care conditions.
- Multidisciplinary healthcare teams can provide better treatment outcomes and quality of care compared to that delivered by individual professionals from a single health discipline.
- Several models of IPE that can be applied to enhance multidisciplinary health care delivery.

## What this study adds

- The process has built capacity for Interprofessional education at UNZA, Ridgeway Campus Schools.
- Seven modules on Chronic Care Conditions have been developed and have since been converted into online training materials for both synchronous and asynchronous teaching and learning.
- An atmosphere of mutual respect and value for other professions was built, in essence igniting a shift in paradigm from profession centrism to a culture of inter-professionalism.
